# Crusted plaques of the scalp after deep brain stimulation surgery

**DOI:** 10.1016/j.jdcr.2026.05.056

**Published:** 2026-06-02

**Authors:** Cayla Vila, Alicia Mizes, Andrew Wensel, Alison Moynihan, Karlo J. Lizarraga

**Affiliations:** a Department of Neurology, University of Rochester, Rochester, New York; b Department of Dermatology, University of Rochester, Rochester, New York; c Department of Neurosurgery, University of Rochester, Rochester, New York

**Keywords:** allergic contact dermatitis, ecthyma, Erosive pustular dermatosis of the scalp, hypertrophic actinic keratosis, impetigo

## Case description

A 77-year-old man with Parkinson’s disease and androgenic alopecia underwent deep brain stimulation (DBS) surgery. The procedure involved two 2 to 3 centimeter frontal scalp incisions for burr hole drilling and lead implantation, followed by subcutaneous tunneling of extension wires under the scalp and behind the ears to the chest for later connection to a pulse generator.

**Fig 1:**
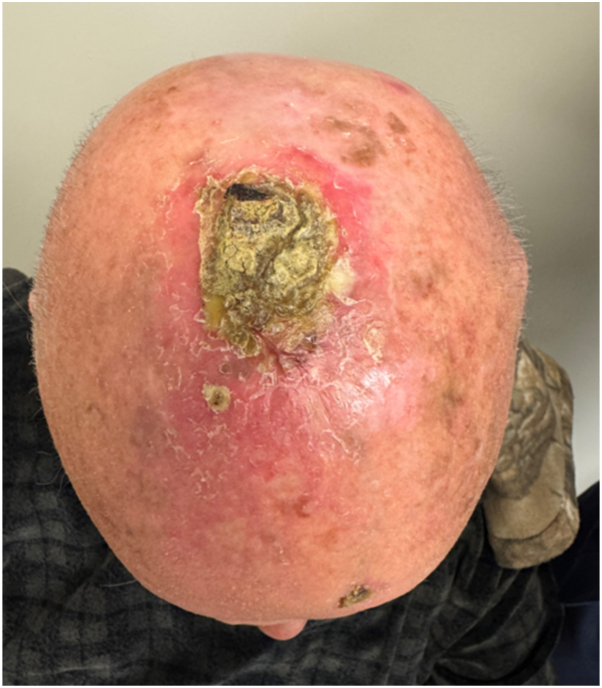
Thick, golden-yellow and brown crusted plaques surrounded by a well-demarcated erythematous patch on the scalp.

Four weeks later, he presented for initial DBS programming. He denied scalp or systemic symptoms. Vital signs were normal. Scalp examination revealed diffuse actinic damage and thick, golden-yellow and brown crusted plaques on the vertex, surrounded by a well-demarcated erythematous patch. The plaques lifted easily, revealing intact, moist epidermis underneath. The surgical incisions were well-healed, without erythematous areas, drainage, or tenderness (Fig 1).

## Question: Which of the following is the most likely diagnosis?

**A.** Erosive pustular dermatosis of the scalp
**B.** Hypertrophic actinic keratosis
**C.** Allergic contact dermatitis
**D.** Ecthyma
**E.** Impetigo

## Impetigo

Post-operative cutaneous complications after DBS surgery include impetigo, cellulitis, wound dehiscence, allergic contact dermatitis (ACD) to implanted hardware, and formation of seromas and hematomas, which may become secondarily infected.^1^, ^2^, ^3^ The frequency of skin-related complications after DBS varies between 1% to 13%.^3^ Infection occurs in approximately 3% of patients with Parkinson’s disease who undergo DBS surgery.^1^ Early recognition is critical to prevent hardware or intracranial involvement necessitating removal of the entire DBS system.^1^

**Fig 2:**
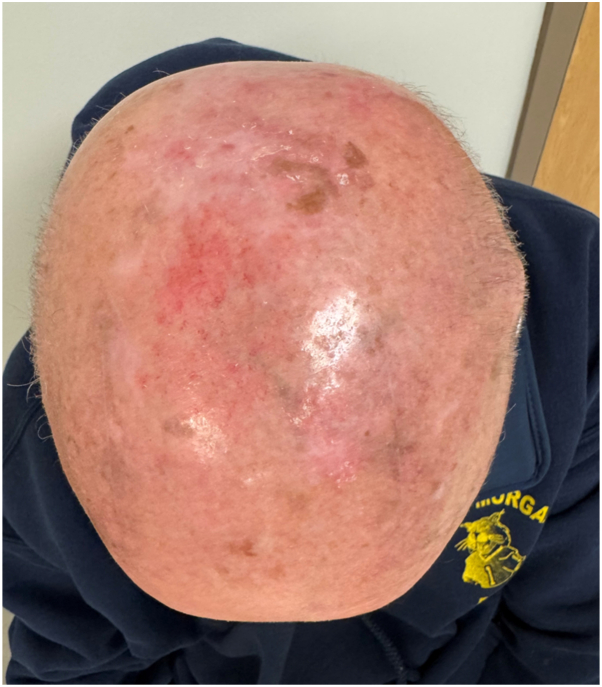
Resolution of the crusted plaques following a 14-day course of antibiotics.

In this patient, the crusted plaques were located on the vertex and relatively distant from the healed frontal incisions. However, the surrounding erythematous patch extended over the subcutaneous extension wires tunneled beneath the scalp. Given the concern for hardware involvement, empiric treatment was initiated with intravenous vancomycin (1.25 g twice daily) and ceftriaxone (2 g twice daily). Complete blood count, C-reactive protein and erythrocyte sedimentation rate were normal. Blood cultures were negative. Computed tomography and magnetic resonance imaging showed no evidence of hardware infection or intracranial involvement. Three days later, superficial cultures from the crusted plaques grew *Streptococcus pyogenes* and methicillin-sensitive *Staphylococcus aureus*. Antibiotic therapy was narrowed to oral cefalexin (500 mg every 6 hours), with complete resolution following a 14-day course (Fig 2).

Erosive pustular dermatosis of the scalp (EPDS) typically affects elderly patients with actinic damage of the scalp, and it may be triggered by local trauma or surgery.^4^ EPDS is a chronic inflammatory condition that produces crusted erosions or pustules that can resemble impetigo. However, EPDS does not respond to antibiotics, and it often persists for months or recurs despite topical corticosteroid therapy.^4^

The frequency of allergic reactions to neuromodulation devices varies between 0.1% to 0.5%.^2^ The most common allergens are polyurethane and nickel.^2^ ACD could present with subacute erythematous patches and plaques, but they are often associated with pruritus.

In this patient, the subacute onset, morphology, cultures identifying clinically relevant gram-positive organisms, and prompt response to antibiotics support a diagnosis of impetigo.^5^ Impetigo and ecthyma are often caused by the same bacteria: *Streptococcus pyogenes* and *Staphylococcus aureus*. Impetigo is localized to the epidermis, whereas ecthyma is characterized by extension into the dermis. Crusted plaques in ecthyma are adherent and their removal reveals superficial ulcers with a depressed base and raised edge, often resulting in scars.

## Conflicts of interest

None disclosed.
